# A deep convolutional neural network for Kawasaki disease diagnosis

**DOI:** 10.1038/s41598-022-15495-x

**Published:** 2022-07-06

**Authors:** Ellen Xu, Shamim Nemati, Adriana H. Tremoulet

**Affiliations:** 1 Department of Pediatrics, University of California San Diego and Rady Children’s Hospital, San Diego, CA USA; 2 Department of Biomedical Informatics, UC San Diego Health, University of California San Diego, La Jolla, CA USA

**Keywords:** Paediatrics, Computational biology and bioinformatics

## Abstract

Kawasaki disease (KD), the most common cause of acquired heart disease in children, can be easily missed as it shares clinical findings with other pediatric illnesses, leading to risk of myocardial infarction or death. KD remains a clinical diagnosis for which there is no diagnostic test, yet there are classic findings on exam that can be captured in a photograph. This study aimed to develop a deep convolutional neural network, KD-CNN, to differentiate photographs of KD clinical signs from those of other pediatric illnesses. To create the dataset, we used an innovative combination of crowdsourcing images and downloading from public domains on the Internet. KD-CNN was then pretrained using transfer learning from VGG-16 and fine-tuned on the KD dataset, and methods to compensate for limited data were explored to improve model performance and generalizability. KD-CNN achieved a median AUC of 0.90 (IQR 0.10 from tenfold cross validation), with a sensitivity of 0.80 (IQR 0.18) and specificity of 0.85 (IQR 0.19) to distinguish between children with and without clinical manifestations of KD. KD-CNN is a novel application of CNN in medicine, with the potential to assist clinicians in differentiating KD from other pediatric illnesses and thus reduce KD morbidity and mortality.

## Introduction

Kawasaki disease (KD) is an acute childhood vasculitis and the leading cause of acquired pediatric heart disease in children, and has been reported in all continents and over 60 countries to date^1,2^. As a missed or delayed treatment can lead to an increased risk of myocardial infarction or death of a child, there is a need for accurate and timely diagnosis of KD to improve patient outcomes^3^. However, KD is often misdiagnosed as it shares clinical findings with other pediatric illnesses^4^. To date, KD remains a disease for which the etiology is unknown and there is no specific test for diagnosis^5^. KD clinical diagnosis is based on criteria established by the American Heart Association (AHA): bilateral conjunctival injection, erythema of lips and oral cavity, polymorphous exanthema, erythema/edema of peripheral extremities, and cervical lymphadenopathy^6^.

In recent years, Convolutional Neural Networks^7^ (CNNs) have achieved state-of-the-art performance on a variety of medical tasks^8–10^. A key factor contributing to the popularity of deep learning in medicine has been the use of scans such as computed tomography (CT) and magnetic resonance imaging (MRI) for radiology^11–14^, and availability of large, annotated datasets for dermatology^15–17^. However, there is a lack of a well-established and large image datasets for KD. We explored techniques to apply CNNs towards medical domains with limited data^18^.

Given that KD clinical findings are visual onsets and can be captured in a photograph, a deep learning image analysis algorithm distinguishing KD from other look-alike illnesses has potential to aid in early diagnosis. In this study, we developed a deep convolutional neural network (KD-CNN) for KD diagnosis through clinical photographs. As clinicians assess clinical signs independently, CNNs were constructed for each individual KD sign. We explored methods to improve model performance given a limited photographic dataset and evaluated the potential of deep learning applied towards a challenging diagnosis.

## Methods

The study was conducted using binary classification differentiating between acute Kawasaki Disease (KD) clinical signs and non-KD images. We used a three-step approach of data acquisition and pre-processing, model development and optimization, and statistical evaluation, in order to construct and validate KD-CNN.

### Data acquisition

**Figure 1:**
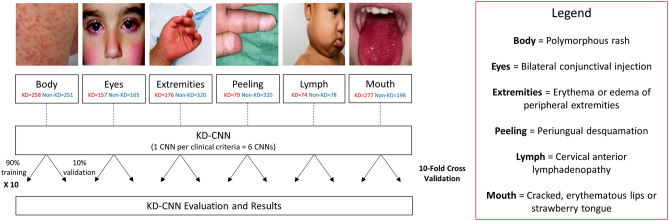
Dataset selection and KD-CNN development diagram. Example images and total number of samples per class, labeled as “KD” and “Non-KD,” are shown for each clinical sign.

The dataset was curated from two primary sources: (1) downloading publicly available retrospective images from the Internet using Google search queries (1510 KD and non-KD images, ~ 74.2% of total dataset) and (2) crowdsourcing from parents of KD patients in collaboration with the KD Foundation (525 KD images, ~ 25.8% of total dataset and ~ 51.3% of KD data). In total, 2,035 images were gathered for the study (1023 KD and 1012 non-KD). The crowdsourcing campaign was launched in August of 2020 through a collaboration with the KD Foundation, who assisted in promotion of the campaign to a large following on social media and KD parent Facebook groups. The project was also presented at the virtual 2020 UCSD KD Parent Symposium with attendees from 17 countries. The guardians/parents of KD patients scanned a QR code to provide informed consent to the KD Foundation and then submitted images online. All photos were uploaded and handled in accordance with the KD Foundation’s guidelines and regulations. We obtained explicit informed consent approved by the UCSD Institutional Review Board for photos in publications, as applicable (Fig. 1). All experimental protocols were approved by the UCSD IRB.

Acute KD patient images for the KD class were curated from both sources, and look-alike disease images for the non-KD class were curated from Source 1 (Internet downloads). Images were then sorted into respective clinical criteria and further adjudicated by a pediatric KD specialist (A.H.T.) to ensure accuracy of labeled data.

### Data pre-processing

We applied data augmentation techniques to create a larger training dataset and improve model invariance. Augments were generated from a range of randomly selected values, instead of fixed-value affine and photographic transformations (e.g., flipping on the horizontal or vertical axis, 90-degree rotations, constant changes in contrast and brightness), which introduced an additional factor of randomness between augments. Each of the following three augments was applied once on the original data, using randomly selected values in the range of valid transformations: rotations from − 90° to 90°, brightness adjustments from 50 to 100% (original brightness), and zooming from 50% zoomed in to 100% (original dimensions).

### Overview of KD-CNN

We developed KD-CNN, an 18-layer convolutional neural network, for classification of KD clinical criteria. KD-CNN takes in a photograph of a patient sign as input and outputs a probability of the sign resembling that of KD versus a look-alike disease. The KD-CNN model development process is shown in Fig. 1.

### Model architecture

There are two main components of the KD-CNN model architecture: a pre-trained VGG-16 model with transfer learning, and additional fully connected layers fine-tuned for classification on the KD and non-KD dataset. To compensate for a small dataset, transfer learning with pre-trained VGG-16 was used to instantiate model weights for efficient training^19^. Initial layers of the network were frozen for low-level feature representation, while the final fully connected layers were used for KD classification feeding into the decision-making step. All models were constructed sequentially using Tensorflow and Keras^20,21^. KD-CNN predictions were compared with the ground truth of labeled classes using categorical cross entropy loss and batch stochastic gradient descent (SGD) with the Adam optimizer^22^. A second additional fully connected layer provided greater degrees of freedom for fine-tuning with KD and non-KD data, hereby referenced as VGG16+.

### Model optimization

Hyperparameters of mini-batch size and steps per epoch, number of epochs, and learning rate were optimized for more efficient model training. We used a small mini-batch size of 4 samples and a default Keras learning rate of 0.001 to trade-off between fast convergence and overshooting minima. To prevent overfitting, we applied regularization steps of early stopping and dropout^23,24^. Early stopping with callbacks of loss and accuracy (maximum of 50 epochs and patience of 5) automatically searched for an optimal halting place during training, instead of manually configurating the number of training epochs. A dropout layer of rate 20% was added before the final VGG16 + fully connected layer to prevent too much co-adaptation. A combination of downsampling the majority class and an adaptive weighted loss function was implemented on a per-criteria basis to help reduce class imbalance^25,26^, e.g., for the Peeling class which had a proportion of 1:4 KD to non-KD images and Extremities class which had 1:2 KD to non-KD images.

Suppose ${\varvec{z}}$ is the predicted output from the model for a true class label $y$ over all classes $j$. Then the weighted loss function $WL\left({\varvec{z}}, y\right)$ is calculated as follows:

1 $$WL\left({\varvec{z}}, y\right)={-\alpha }_{y}\mathrm{log}\left(\frac{\mathrm{exp}\left({z}_{y}\right)}{\sum_{j=1}^{2}\mathrm{exp}\left({z}_{j}\right)}\right),$$

where the weighting factor ${\alpha }_{y}$ is inversely proportional to the effective number of samples per class. The weighting factor with ${n}_{y}$ number of samples for the class $y$ and $N$ total number of samples is calculated as:

2 $${\alpha }_{y}=1-\frac{{n}_{y}}{N},$$

where $y$ is either the KD or non-KD class.

### Statistical methods and evaluation

We evaluated KD-CNN performance using tenfold cross validation, typically a less biased and less optimistic performance estimate compared to a single realization of a train-test split^27^. Each sample was used in nine separate folds for training and one time for testing (90–10 train-test split), and performance was measured on previously unseen samples during testing. Other methods used to evaluate model performance were area under the receiver operating characteristic curves (AUC)^28^, confusion matrices^29^, and true class probability (TCP) charts^30^. TCP charts plot a distribution of raw probability predictions by removing the final softmax activation, instead of the typical maximum class probability (MCP) output of binary classifications. Samples which were incorrectly classified with high probability (> 70% threshold) based on TCP were flagged for human review to further examine misclassified images. From the confusion matrix, additional metrics not dependent on prevalence used to evaluate model performance were sensitivity, specificity, and diagnostic odds ratio (DOR)^31^.

## Results

The KD-CNN dataset gathered from Internet sources and crowdsourcing is shown in Table 1.

**Table 1:** Number of samples per clinical criteria in the KD-CNN dataset (prior to augmentation).

Clinical criteria	Tag	KD	Non-KD^a^	Total
1. Polymorphous rash	Body	258	251	509
2. Bilateral conjunctival injection	Eyes	157	165	322
3. Erythema of peripheral extremities	Extremities	176	320	496
4. Peeling of peripheral extremities	Peeling	79	320	399
5. Cervical anterior lymphadenopathy	Lymph	74	78	152
6. Changes in the lips and oral cavity	Mouth	277	198	475
Total		1023	1012	2035

Examples of search terms for Internet queries included “Kawasaki disease strawberry tongue,” “Kawasaki disease red eye,” “Kawasaki disease anterior cervical lymphadenopathy,” “Kawasaki Disease rash” for KD data and “hand foot mouth disease,” “scarlet fever,” “fifth disease,” “toxic shock syndrome,” “staphylococcal scalded skin syndrome” for non-KD data. Erythema and peeling are separated as clinical criteria to distinguish acute KD and subacute progressions for early diagnosis. Crowdsourced data was from 14 countries: US, France, Croatia, Slovakia, Albania, Philippines, Denmark, Canada, Mexico, UK, Indonesia, New Zealand, Australia, and Brazil.

^a^The same datasets were used for both Erythema of peripheral extremities and peeling of peripheral extremities for non-KD, thus leading to a total of 1012 unique images.

**Figure 2:**
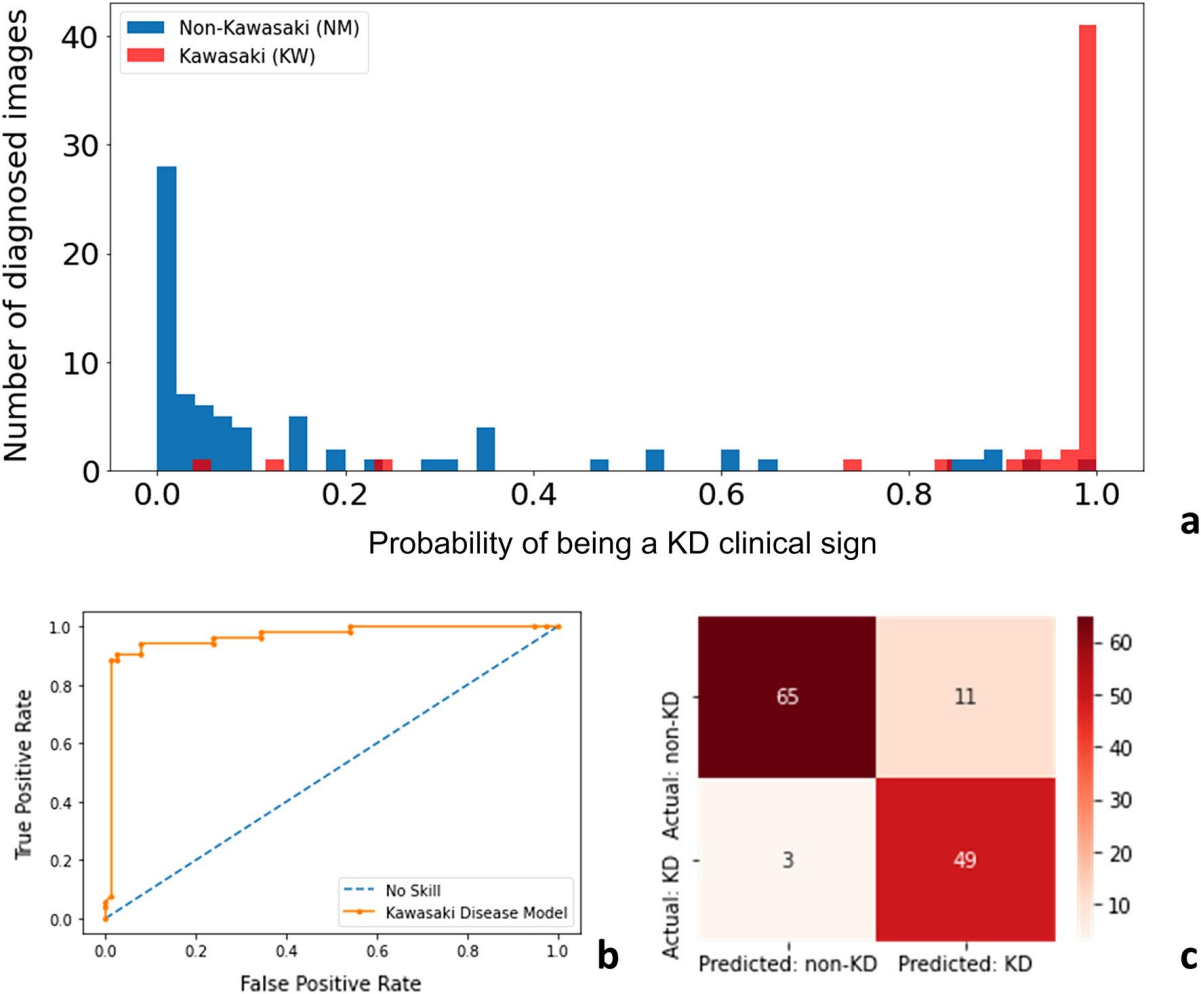
Examples of types of model evaluation used in each fold of cross validation: (**a**) true class probability chart, (**b**) area under the curve of receiver operating characteristic, (**c**) confusion matrix.

We built the KD-CNN model and evaluated optimization techniques through the statistical methods shown in Fig. 2. Each clinical sign model was constructed trained and evaluated independently.

From the tenfold cross validation testing results, KD-CNN achieved a median AUC of 0.90 (IQR 0.10) with a sensitivity of 0.80 (IQR 0.18) and specificity of 0.85 (IQR 0.19) to distinguish between children with and without clinical manifestations of KD (Table 2). Based on the Diagnostic Odds Ratio, which measures the effectiveness of a diagnostic test independent from prevalence where a higher score is indicative of better performance (> 1 is considered a useful test), the performance of individual sign models (in decreasing order) is Extremities (DOR 136.52), Eyes (55.68), Mouth (35.37), Lymph (19.49), Body (12.28), and Peeling (9.53).

**Table 2:** Summary of tenfold cross validation results across KD clinical criteria.

	Body	Eyes	Extremities	Peeling	Lymph	Mouth	Median
Accuracy	0.75 (0.05)	0.84 (0.10)	0.90 (0.05)	0.73 (0.08)	0.79 (0.08)	0.84 (0.05)	0.82 (0.14)
Sensitivity	0.77 (0.13)	0.79 (0.22)	0.78 (0.19)	0.7 (0.19)	0.77 (0.13)	0.88 (0.07)	0.80 (0.18)
Specificity	0.72 (0.12)	0.89 (0.07)	0.95 (0.05)	0.73 (0.29)	0.79 (0.17)	0.78 (0.11)	0.85 (0.19)
AUC	0.83 (0.07)	0.92 (0.05)	0.97 (0.04)	0.79 (0.09)	0.85 (0.06)	0.91 (0.04)	0.90 (0.10)
DOR	12.28	55.68	136.52	9.53	19.49	35.37	27.43

## Discussion

We developed a convolutional neural network that can distinguish with high sensitivity and specificity between the clinical signs of KD and signs of other pediatric illnesses through patient photographs. KD-CNN is the first application of deep learning to the diagnosis of KD, achieving an overall AUC of 0.90. While deep learning has been previously investigated for the detection of skin disorders using photographs^15–17^, there are few studies applying deep learning for clinical diagnosis of pediatric diseases. A few studies have been conducted for neural networks for KD assessment and prediction^32–34^. To our knowledge, our study is the first to develop image-based deep learning methods for KD.

KD-CNN utilizes photographs of patient clinical features, which can be easily taken on a smartphone device, to classify KD from look-alike diseases. We used an innovative combination of Internet downloads and crowdsourcing from parents of KD patients for data collection. Given the lack of a pre-existing dataset and publicly available images on the Internet alone, we leveraged unique crowdsourcing methods to incorporate data from a variety of different geographical locations and generalize across a larger population. To improve model training, we applied pre-training and transfer learning to inherit weights from VGG-16, and added second fully connected layer (VGG16 + architecture) to allow greater fine-tuning on the KD and non-KD dataset. Additional layers beyond VGG16 + did not yield significant improvement in performance, most likely due to the limited data available to train models of increasing complexity and the advent of overfitting. Since some clinical signs included a greater proportion of non-KD images than KD, an adaptive weighted loss function was created to mitigate class imbalance, through applying class weights proportional to the relative number of samples per class.

There are both strengths and limitations to this study. A primary limitation is the size of the dataset, given the absence of a well-established and pre-existing image database for KD. The uncommon nature of the disease prevented collection of a high volume of images, such as thousands of samples per class typical for deep learning studies. Furthermore, despite the geographical diversity of crowdsourced data, the exact demographic information and breakdown of the patient population such as race was not collected, which limited our ability to assess association of patient characteristics with model predictions. Additional testing with well-characterized patient data from, as well as greater investigation into potential algorithmic bias, will help further validate KD-CNN during our next stage of research. Development of a composite score integrating multiple patient photographs, demographic information, and initial laboratory values would also be worth exploring in future work.

## Conclusion

KD-CNN is a novel application of CNN image classification for KD clinical sign diagnosis. This study highlights methods of data crowdsourcing and deep learning methodologies towards new applications of AI and provides support that a deep learning algorithm can help distinguish between photographs of the clinical signs of KD and other pediatric illnesses.

## Data Availability

The de-identified datasets (images and pseudocode) used in this study are available from the corresponding author upon reasonable request.
